# A case analysis of Gitelman syndrome^1^ complicated with Sjögren’s disease

**DOI:** 10.3389/fmed.2026.1842176

**Published:** 2026-06-19

**Authors:** Yuqi Tang, Sen Tian, Cong Xia, Yan Zhang, Qiaoding Dai

**Affiliations:** The First Affiliated Hospital of Zhejiang Chinese Medical University, Hangzhou, China

**Keywords:** comorbidity, Gitelman syndrome, hypokalemia, Sjögren’s disease, SLC12A3 gene

## Abstract

**Background:**

Sjögren’s disease (an autoimmune exocrinopathy) and Gitelman syndrome (an autosomal recessive renal tubulopathy caused by SLC12A3 mutations) both manifest with hypokalemia. Their coexistence can significantly complicate differential diagnosis.

**Methods:**

A 62-years-old female presented with fatigue, dry mouth, and refractory hypokalemia. Immunological testing (positive antinuclear and anti-centromere protein B antibodies) and a labial gland biopsy confirmed Sjögren’s disease. However, her severe hypokalemia was disproportionate to Sjögren’s-induced renal tubular acidosis alone. Genetic analysis revealed compound heterozygous pathogenic mutations in SLC12A3: c.1196G > A (p.Arg399His) and c.1732G > A (p.Val578Met), confirming concurrent Gitelman syndrome.

**Results:**

Combined therapy with potassium supplementation and hydroxychloroquine successfully resolved symptoms and stabilized serum potassium levels.

**Conclusion:**

In Sjögren’s disease patients with refractory hypokalemia, underlying hereditary renal tubular disorders should be suspected. Combining immunological evaluation with genetic testing is crucial to ensure accurate diagnosis and optimize management.

## Introduction

Gitelman syndrome (GS) is an autosomal recessive renal tubular disorder caused by mutations in the SLC12A3 gene located on chromosome 16q13. This gene encodes the thiazide-sensitive sodium-chloride cotransporter (NCC) (1), estimated prevalence 1–25 per 40,000 (1). Functional impairment of NCC leads to defective reabsorption of sodium, chloride, potassium, and magnesium in the distal convoluted tubules, clinically presenting as hypokalemia, hypomagnesemia, hypocalciuria, and metabolic alkalosis (2). Sjögren’s disease (SJD) is a chronic autoimmune disorder primarily affecting exocrine glands such as the salivary and lacrimal glands. It is characterized by symptoms like dry mouth and dry eyes resulting from lymphocytic infiltration, and predominantly occurs in middle-aged women (3, 4).

It is noteworthy that both GS and SJD can cause symptoms such as fatigue, muscle weakness, and electrolyte disturbances, leading to overlapping clinical presentations (5). In recent years, several case reports have suggested that GS patients may coexist with autoimmune diseases such as SJD, which increases diagnostic complexity (6, 7). This article presents a case of refractory hypokalemia as the initial manifestation, subsequently diagnosed as GS complicated with SJD through genetic testing and labial gland biopsy. A literature review is also included, aiming to enhance clinicians’ awareness of the diagnosis and management of such comorbidities.

## Case report

The patient, a 62-years-old female, was admitted due to “recurrent hypokalemia for 2 years.” Two years prior, hypokalemia (mild to moderate, with an estimated serum potassium level of 3.0–3.4 mmol/L based on the outpatient summary) was detected during a physical examination, accompanied by fatigue. No typical symptoms of dry mouth or dry eyes were present at that time. She had been on long-term oral potassium chloride sustained-release tablets (1.5 g/day) treatment, but her serum potassium levels remained recurrently low. Recently, she developed dry mouth without significant dry eyes. Her past medical history included chronic gastritis, atopic dermatitis, colon polypectomy, and pulmonary nodules. She denied any history of hypertension or diabetes mellitus. She also had a history of leukopenia for 2 years and was on long-term treatment with Leucogen tablets.

1. Preliminary examination and diagnosis of SJD

After admission, electrolyte tests showed: serum potassium: 2.52 mmol/L (reference range: 3.5–5.3 mmol/L), serum magnesium: 0.86 mmol/L (reference range: 0.75–1.02 mmol/L). Arterial blood gas analysis indicated metabolic alkalosis. Urinary chloride concentration was elevated. Complete blood count revealed persistently low white blood cell counts.

**FIGURE 1:**
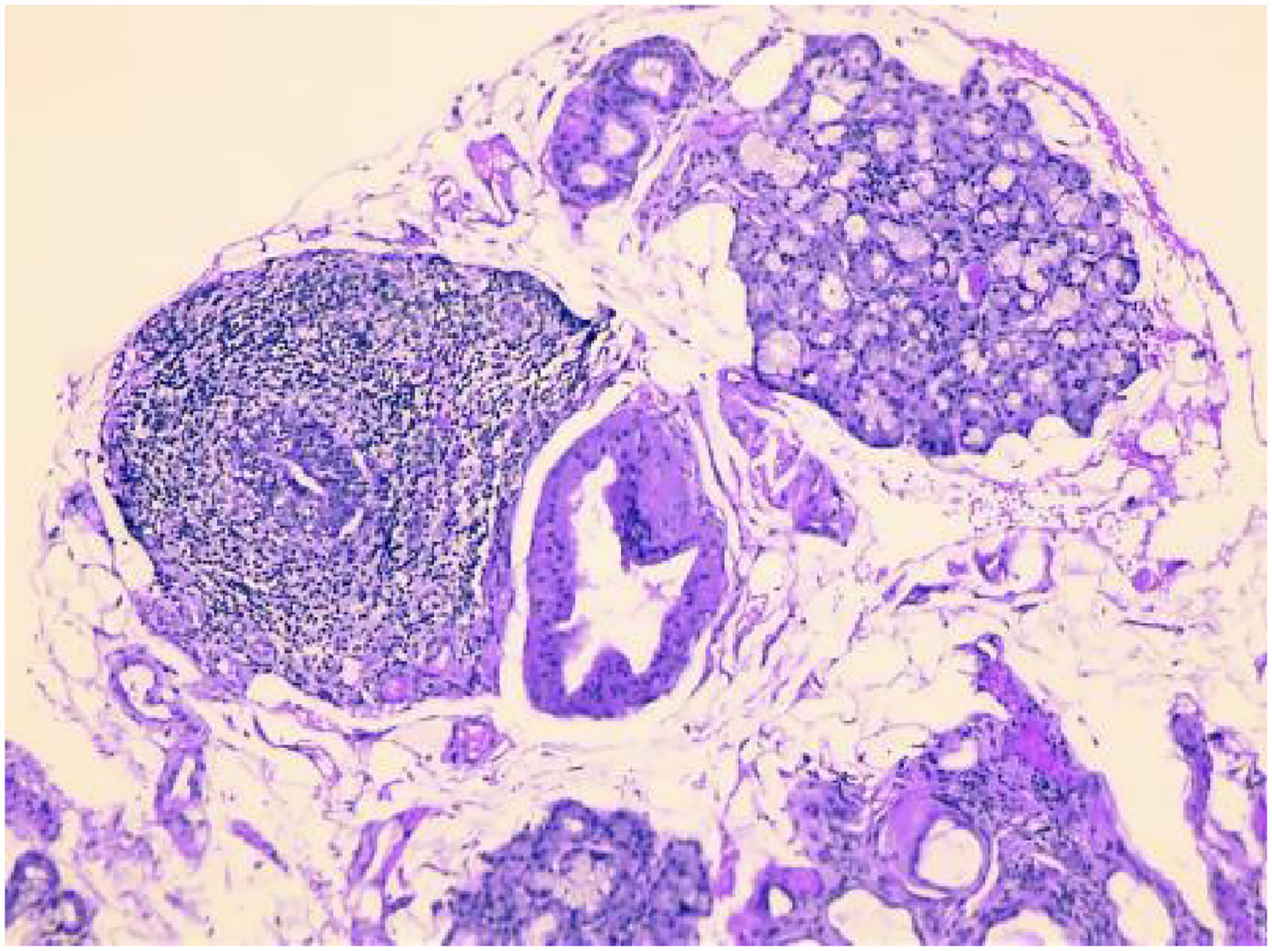
Histopathological examination of the labial salivary gland biopsy revealed significant focal lymphocytic infiltration.

Given the patient’s symptom of dry mouth, Sjögren’s disease (SJD) was clinically suspected. Subsequent rheumatological and immunological tests were performed: antinuclear antibodies (ANA) was positive (1:640), and anti-centromere protein B (anti-CENP-B) antibodies were positive. A labial gland biopsy showed acinar atrophy and lymphocytic infiltration (1 focus/4mm^2^, with >50 lymphocytes per focus) (Figure 1), meeting the diagnostic criteria of the 2016 ACR/EULAR classification criteria for Sjögren’s disease (8).

2. Stepwise investigation into the cause of hypokalemia

The clinical approach to diagnosing and managing hypokalemia typically follows a logical progression, moving from identifying the route of potassium loss to analyzing hormone levels. The initial step involves monitoring urinary potassium levels to differentiate the source of loss: a urinary potassium level below 20 mmol/L suggests extrarenal causes such as inadequate intake, gastrointestinal loss (e.g., diarrhea), or an intracellular shift (e.g., thyrotoxic periodic paralysis). Conversely, a level above 20 mmol/L indicates renal potassium wasting, necessitating further investigation (9). For these patients, blood pressure assessment is critical. In cases with elevated blood pressure, testing renin and aldosterone levels helps distinguish between conditions like primary aldosteronism, renal artery stenosis, or Cushing’s syndrome (10). If blood pressure is normal or low, blood gas analysis is required to identify the underlying cause based on the presence of acidosis (e.g., renal tubular acidosis) or alkalosis (e.g., diuretic use, Bartter syndrome, or Gitelman syndrome).

Although the patient had a clear diagnosis of SJD, and SJD can be complicated by renal tubular acidosis leading to hypokalemia, her arterial blood gas analysis indicated only metabolic alkalosis without evidence of hyperchloremic acidosis. Urine pH did not suggest significant acidification impairment, and renal function along with urinary electrolytes did not support typical renal tubular acidosis. Therefore, SJD alone could not adequately explain her persistent and refractory hypokalemia.

To further clarify the etiology of hypokalemia, and considering the differential diagnosis of hypokalemia, the possibility of an inherited renal tubular disorder was taken into account. Genetic testing for hypokalemia-related genes was performed (the testing panel included SLC12A3 and 41 other genes). The results revealed two heterozygous pathogenic variants in the SLC12A3 gene: c.1196G > A (p.Arg399His) (classified as a variant of uncertain significance per ACMG criteria: PM2_Supporting, PM3_Supporting, PP3) and c.1732G > A (p.Val578Met) (classified as likely pathogenic per ACMG criteria: PM3_Strong, PP3_Moderate). This molecular diagnostic finding is consistent with Gitelman syndrome (11, 12).

The patient’s genetic testing results are as follows (Tables 1, 2):

**TABLE 1:** Pathogenic or likely pathogenic variants highly consistent with the disease.

Gene/ transcript	Physical location	Gene region	Variant information	Zygosity	Pathogenicity classification	Inheritance	Disease/ phenotype	RS number	Family source	Reference
*SLC12A3* NM_00112 6108.2	chr16:56 913000	Exon 10	c.1196G > A (p.Arg399His)	Heterozygous	Variant of uncertain significance	AR	Gitelman syndrome	rs 13306 668	–	(1)
*SLC12A3* NM_00112 6108.2	chr16:56 918023	Exon 14	c.1732G > A p.Val578Met	Heterozygous	Likely Pathogenic	AR	Gitelman syndrome	rs13932 9616	–	(1, 2)

**TABLE 2:** Sanger sequencing results.

Analyzed sample	Analysis result	*SLC12A3*	chrl 6:56913000	c.1196G > A	p.Arg399His
Patient	Heterozygous variant	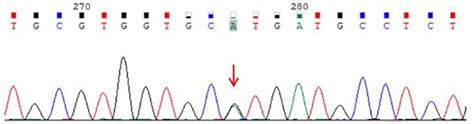			
**Analyzed sample**	**Analysis result**	***SLC12A3***	**chrl 6:56918023**	**c.1732G > A**	**p.Val578Met**
Patient	Heterozygous variant	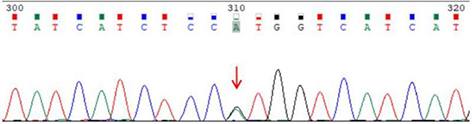			

**FIGURE 2:**
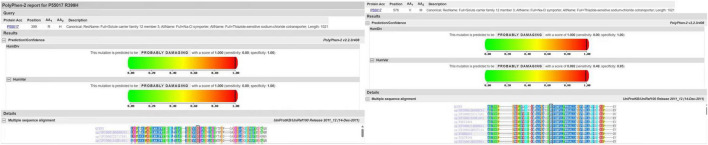
PolyPhen-2 bioinformatic analysis of SLC12A3 variants.

Subsequently, we used Polyphen-2 software to analyze these two mutations, and the prediction results indicated that both are pathogenic (Figure 2).

3. Final diagnosis and treatment follow-up

Final diagnosis: ① Gitelman syndrome; ② Sjögren’s disease.

Treatment plan: while continuing oral potassium supplementation (1.5 g, once daily) to correct electrolyte imbalances, hydroxychloroquine sulfate (0.2 g, twice daily) was added for immunomodulation. The patient received long-term traditional Chinese medicine treatment in the later stage, with prescriptions including *Adenophora stricta*, *Polygonatum odoratum*, *Dendrobium nobile*, etc., (13).

**FIGURE 3:**
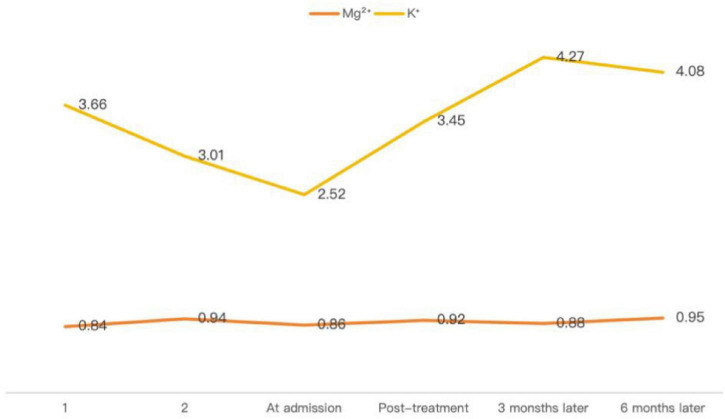
Serial measurements of serum electrolytes before and after therapy.

Follow-up: the patient was regularly followed up in the Rheumatology and Nephrology departments after discharge. The patient’s serum potassium level normalized after 1 week of treatment. Her fatigue symptoms significantly improved, and serum potassium levels remained stable (Figure 3).

## Discussion

This case illustrates the clinical complexity of the coexistence of GS and SJD. The coexistence of GS and SJD may not be coincidental. Therefore, for patients exhibiting features of both autoimmune disease and refractory electrolyte disturbances, a simplistic “unitary” attribution should be avoided. Although both SJD and GS can cause hypokalemia, their mechanisms differ (Table 3). Studies indicate that SJD often leads to potassium loss via renal tubular acidosis, whereas GS results from impaired sodium-chloride reabsorption in the distal convoluted tubule. When these conditions coexist, symptom overlap can easily lead to underdiagnosis of GS. Furthermore, chronic electrolyte disturbances and activation of the renin-angiotensin-aldosterone system (RAAS) may impact immune homeostasis, potentially increasing the risk of autoimmune diseases (14, 15). On the other hand, SJD can be complicated by tubulointerstitial nephritis, which theoretically may exacerbate potassium and magnesium loss, creating a vicious cycle (16, 17).

**TABLE 3:** Differential diagnosis between Gitelman syndrome and SJD-related RTA.

Feature	Gitelman syndrome (GS)	SJD-related RTA
Acid-base balance	Metabolie alkalosis	Metabolic acidosis
Serum chloride	Hypochloremia	Hyperchloremia (often)
Urinary calcium	Hypocalciuria	Normal or high
Pathogenesis	*SLC12A3* mutations	Distal tubule acidification defect

In this case, the immunological profile revealed positivity for both ANA and anti-CENP-B antibodies. While anti-CENP-B is classically associated with systemic sclerosis, it is identified in approximately 5%–10% of SJD patients, typically defining a distinct clinical subgroup characterized by a higher prevalence of Raynaud’s phenomenon and exocrine gland dysfunction. A comprehensive management strategy is required in treatment: SJD necessitates the use of immunomodulators such as hydroxychloroquine to control immune dysregulation, along with regular monitoring for systemic involvement (18, 19); GS requires lifelong electrolyte (potassium, magnesium) supplementation (20, 21). Multidisciplinary collaboration (between nephrology and rheumatology) is crucial for the long-term management of such patients (22, 23).

To better contextualize the uniqueness of this case, we expanded our literature review to include previously reported GS cases across different age groups, particularly late-onset presentations in patients over 60 years old. While GS is traditionally diagnosed in childhood or adolescence, several elderly cases have been documented worldwide. For instance, increasing clinical literature has documented the occurrence of this condition in advanced age; in 2023, Practical Geriatrics reported several cases of “elderly-onset Gitelman syndrome” (24). Expanding on this, a landmark study published in Aging-US in 2025 identified the oldest GS patient globally to date–an 83-years-old female–explicitly noting that GS remains frequently overlooked in the elderly and emphasizing its inclusion in the differential diagnosis of unexplained electrolyte disturbances across all age groups (25). In alignment with these findings, an independent report characterized a 60-years-old female diagnosed with GS who manifested profound electrolyte disturbances, including a serum potassium of 2.91 mmol/L and serum magnesium of 0.6 mg/dL (26). In comparison to these previously reported elderly cases, our patient’s compound heterozygous mutations, c.1196G > A (p.Arg399His) and c.1732G > A (p.Val578Met), appear to manifest a particularly prolonged subclinical phase. To our knowledge, this specific combination of variants has not been previously characterized in a diagnostic context for patients over 60 years of age. This broader comparison reinforces that the phenotypic expression of GS is highly heterogeneous, with the onset age potentially dictated by the specific combination of SLC12A3 alleles and the presence of secondary metabolic or immunological triggers.

Notably, the patient was diagnosed at the age of 62, which is significantly later than the typical pediatric onset of GS. This clinical presentation suggests a potentially milder genotype-phenotype correlation associated with the identified compound heterozygous mutations in SLC12A3: c.1196G > A (p.Arg399His) and c.1732G > A (p.Val578Met). Although *in silico* tools like PolyPhen-2 predict these variants to be damaging, the delayed onset indicates that the NCC protein likely retained sufficient residual function to maintain electrolyte balance during the patient’s earlier life. The transition from a subclinical state to overt clinical symptoms at age 62 was likely triggered by the cumulative effect of age-related decline in renal reserve and the additional immunological insult to the renal tubules following the onset of Sjögren’s disease. This “dual-hit” mechanism explains why the genetic predisposition only manifested clinically in the presence of secondary acquired factors later in life.

It challenges the traditional notion that “hereditary renal tubular diseases inevitably manifest in early childhood and are diagnosed promptly.” It clearly highlights that GS, as a disease with considerable phenotypic heterogeneity, may present with mild, non-specific initial symptoms, or symptoms overlapping with other acquired conditions (such as autoimmune diseases). This overlap can lead to prolonged diagnostic delay or oversight until adulthood or old age, when the condition is discovered due to complications or incidental findings. It is noteworthy that the association between GS and autoimmune disorders is not limited to Sjögren’s disease. Emerging evidence indicates that GS may coexist with a broader spectrum of autoimmune conditions, most notably autoimmune thyroid diseases (AITD), such as Graves’ disease and Hashimoto’s thyroiditis (27). The recurrent finding of GS alongside various autoimmune manifestations supports the hypothesis that the chronic electrolyte imbalances and neuroendocrine disturbances inherent in GS may create a systemic milieu predisposed to immune dysregulation. By broadening the clinical context of these associations, clinicians can better recognize and manage the complex multisystem involvement in patients with hereditary tubulopathies.

Although the coexistence of GS and SJD is rare, we propose that the chronic activation of the RAAS in GS patients may play a role in the breakdown of immune homeostasis. Accumulating evidence suggests that Angiotensin II, the primary effector of the RAAS, functions as a potent pro-inflammatory modulator by regulating the balance between Th17 and regulatory T cells (Tregs) (28). Furthermore, the chronic hypomagnesemia observed in GS has been linked to increased systemic inflammation (29). Thus, the persistent hyperreninemic hyperaldosteronism in GS might provide a permissive environment for the development or exacerbation of autoimmune responses in SJD.

In clinical practice, the most critical biochemical hallmark to distinguish GS from Sjögren’s-related renal tubular acidosis (RTA) is the type of acid-base disturbance. While GS is characteristically associated with metabolic alkalosis and hypochloremia, SJD-related RTA typically presents with normal anion gap metabolic acidosis. In this case, although the patient had established SJD, the presence of metabolic alkalosis rather than acidosis was a key clue that led us to suspect a concurrent genetic tubulopathy, eventually confirmed by genetic testing.

The core takeaway from this case is that: in patients with Sjögren’s disease presenting with refractory hypokalemia that is unresponsive to conventional treatment, early genetic testing should be considered to identify potential underlying hereditary renal tubular disorders. Clinical diagnosis should adhere to a stepwise, layered analytical approach. Early integration of genetic testing with immunological evaluation is essential to establish a comorbid diagnosis, thereby enabling the formulation of a personalized treatment plan and treatment protocols of traditional Chinese medicine [particularly utilizing traditional medicine and natural products (30)] to improving patient prognosis. As a rare case of GS diagnosed at an advanced age, this report expands the age spectrum of GS onset and provides important empirical evidence for the lifelong management of this disease. We advocate for heightened awareness regarding screening for genetic etiologies in cases of atypical (31), refractory electrolyte disturbances within adult, particularly geriatric, internal medicine, rheumatology, and nephrology practices. Future research should further investigate the epidemiological characteristics, clinical phenotypes, and factors contributing to delayed diagnosis of GS in adult and elderly populations. It should also aim to clarify the true incidence and complete clinical picture of GS-SJD comorbidity and delve deeper into potential shared pathophysiological mechanisms. This will provide a more robust theoretical foundation for the precise diagnosis, treatment, and systematic management of such patients (32).

## Data Availability

The single nucleotide polymorphism data supporting the findings of this study have been deposited in the NCBI dbSNP repository with the accession numbers rs13306668 and rs139329616. The data can be accessed directly via the following URLs: https://www.ncbi.nlm.nih.gov/snp/rs13306668 and https://www.ncbi.nlm.nih.gov/snp/rs139329616.
