# Supplementary material for: A case analysis of Gitelman syndrome complicated with Sjögren’s disease
Source: Front Med (Lausanne). 2026 Jun 19;13:1842176. doi: 10.3389/fmed.2026.1842176 (PMC13327935; doi:10.3389/fmed.2026.1842176)

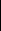

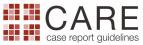

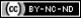

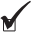
**CARE** **Checklist** **of** **information** **to** **include** **when** **writing** **a** **case** **report**

**Topic**

**Title**

**Key** **Words**

**Abstract**

**(no** **references)**

**Introduction**

**Patient** **Information**

**Clinical** **Findings** **Timeline**

**Diagnostic** **Assessment**

**Therapeutic** **Intervention**

**Follow-up** **and** **Outcomes**

**Discussion**

**Patient** **Perspective** **Informed** **Consent**


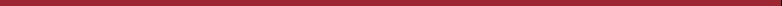


**Item** **Checklist** **item** **description** **Reported** **on** **Line**

**1** The diagnosis or intervention of primary focus followed by the words “case report” . . . . . . . . . . . . . . . . . . . . . . . . . . 1

**2** 2 to 5 key words that identify diagnoses or interventions in this case report, including "case report" . . . 27 **3a** Introduction: What is unique about this case and what does it add to the scientific literature? . . . . . . . . . . . . . . . . 29-44 **3b** Main symptoms and/or important clinical findings . . . . . . . . . . . . . . . . . . . . . . . . . . . . . . . . . . . . . . . . . . . . . . . . . . . . . . . 49-68 **3c** The main diagnoses, therapeutic interventions, and outcomes . . . . . . . . . . . . . . . . . . . . . . . . . . . . . . . . . . . . . . . . . . . 69-95 **3d** Conclusion—What is the main “take-away” lesson(s) from this case? . . . . . . . . . . . . . . . . . . . . . . . . . . . . . . . . . . . . . 141-158

**4** One or two paragraphs summarizing why this case is unique (**may** **include** **references**) . . . . . . . . . . . . 103-125 **5a** De-identified patient specific information. . . . . . . . . . . . . . . . . . . . . . . . . . . . . . . . . . . . . . . . . . . . . . . . . . . . 47-55 **5b** Primary concerns and symptoms of the patient. . . . . . . . . . . . . . . . . . . . . . . . . . . . . . . . . . . . . . . . . . . . . . . . . . . . . 30-44 **5c** Medical, family, and psycho-social history including relevant genetic information . . . . . . . . . . . . . . . . . 56-58 **5d** Relevant past interventions with outcomes . . . . . . . . . . . . . . . . . . . . . . . . . . . . . . . . . . . . . . . . . . . . . . . . . . . . . . . . 50-51

**6** Describe significant physical examination (PE) and important clinical findings. . . . . . . . . . . . . . . . . . . . . . . 75-82

**7** Historical and current information from this episode of care organized as a timeline . . . . . . . . . . . . . . . 61-98 **8a** Diagnostic testing (such as PE, laboratory testing, imaging, surveys). . . . . . . . . . . . . . . . . . . . . . . . . . . . . . . 75-82 **8b** Diagnostic challenges (such as access to testing, financial, or cultural) . . . . . . . . . . . . . . . . . . . . . . . . . . . . . 69-74 **8c** Diagnosis (including other diagnoses considered) . . . . . . . . . . . . . . . . . . . . . . . . . . . . . . . . . . . . . . . . . . . . . . . . . 92 **8d** Prognosis (such as staging in oncology) where applicable . . . . . . . . . . . . . . . . . . . . . . . . . . . . . . . . . . . . . . . . . 96-98 **9a** Types of therapeutic intervention (such as pharmacologic, surgical, preventive, self-care) . . . . . . . . . . . . . . . . . . . . 93-95 **9b** Administration of therapeutic intervention (such as dosage, strength, duration) . . . . . . . . . . . . . . . . . . . . . . . . . . . . . 93-95 **9c** Changes in therapeutic intervention (with rationale) . . . . . . . . . . . . . . . . . . . . . . . . . . . . . . . . . . . . . . . . . . . . . . . . . . . . 93-95

**10a** Clinician and patient-assessed outcomes (if available) . . . . . . . . . . . . . . . . . . . . . . .. . . . . . . . . . . . . . . . . . . . . . . . . . . . 95-98 **10b** Important follow-up diagnostic and other test results . . . . . . . . . . . . . . . . . . . . . . . . . . . . . . . . . . . . . . . . . . . . . . . . . . . . 92-98 **10c** Intervention adherence and tolerability (How was this assessed?) . . . . . . . . . . . . . . . . . . . . . . . . . . . . . . . . . . . . . . . . . 99-101 **10d** Adverse and unanticipated events . . . . . . . . . . . . . . . . . . . . . . . . . . . . . . . . . . . . . . . . . . . . . . . . . . . . . . . . . . . . . . . . . . . none **11a** A scientific discussion of the strengths AND limitations associated with this case report . . . . . . . . . . . . . . . . . . . . . . . 141-157 **11b** Discussion of the relevant medical literature **with** **references**. . . . . . . . . . . . . . . . . . . . . . . . . . . . . . . . . . . . . . . . . . 161-232 **11c** The scientific rationale for any conclusions (including assessment of possible causes) . . . . . . . . . . . . . . . . . . . . . . . . 75-82 **11d** The primary “take-away” lessons of this case report (without references) in a one paragraph conclusion . . . . . . . 8-24

**12** The patient should share their perspective in one to two paragraphs on the treatment(s) they received . . . . . . . . 99-101

**13** Did the patient give informed consent? Please provide if requested . . . . . . . . . . . . . . . . . . . . . . . . . . . . . . . . . . . . . . **Yes**
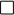
 **No**
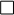

Supplement: Supplementary file 1 [file Table_1.DOCX]
